# Discharge Enhancement in a Triple-Pipe Heat Exchanger Filled with Phase Change Material

**DOI:** 10.3390/nano12091605

**Published:** 2022-05-09

**Authors:** Yongfeng Ju, Roohollah Babaei-Mahani, Raed Khalid Ibrahem, Shoira Khakberdieva, Yasir Salam Karim, Ahmed N. Abdalla, Abdullah Mohamed, Mustafa Z. Mahmoud, Hafiz Muhammad Ali

**Affiliations:** 1 Faculty of Electronics Information Engineering, Huaiyin Institute of Technology, Huai’an 223003, China; dramaidec@gmail.com; 2 Department of Chemical Engineering, Brunel University London, Kingston Lane, Uxbridge UB8 3PH, UK; roohollah.babaei-mahani@brunel.ac.uk; 3 Department of Medical Instrumentation Engineering Techniques, Al-Farahidi University, Baghdad 10015, Iraq; raed.k.ibrahim@uoalfarahidi.edu.iq; 4 Department of Scientific Affairs, Tashkent State Pedagogical University Named after Nizami, Tashkent 100185, Uzbekistan; shoira.khakberdieva.uzb@mail.ru; 5 Department of Pharmacy, Al-Manara College for Medical Sciences, Maysan 62001, Iraq; yasirsalamkarim@uomanara.edu.iq; 6 Department of Pharmacy, Al-Nisour University College, Baghdad 10081, Iraq; 7 Research Centre, Future University in Egypt, New Cairo 11745, Egypt; mohamed.a@fue.edu.eg; 8 Department of Radiology and Medical Imaging, College of Applied Medical Sciences, Prince Sattam Bin Abdulaziz University, Al-Kharj 11942, Saudi Arabia; m.alhassen@psau.edu.sa; 9 Faculty of Health, University of Canberra, Canberra 2600, Australia; 10 Mechanical Engineering Department, King Fahd University of Petroleum and Minerals, Dhahran 31261, Saudi Arabia; 11 Interdisciplinary Research Center for Renewable Energy and Power Systems (IRC-REPS), King Fahd University of Petroleum and Minerals, Dhahran 31261, Saudi Arabia

**Keywords:** discharge, phase change materials, triple-pipe heat exchanger, performance enhancement

## Abstract

This study aims to study the discharging process to verify the influence of geometry modifications and heat transfer flow (HTF) patterns on the performance of a vertical triplex-tube latent heat container. The phase change material (PCM) is included in the middle tube, where the geometry is modified using single or multi-internal frustum tubes instead of straight tubes to enhance the discharging rate. The effects of the HTF flow direction, which is considered by the gravity and opposite-gravity directions, are also examined in four different cases. For the optimal geometry, three scenarios are proposed, i.e., employing a frustum tube for the middle tube, for the inner tube, and at last for both the inner and middle tubes. The effects of various gap widths in the modified geometries are investigated. The results show the advantages of using frustum tubes in increasing the discharging rate and reducing the solidification time compared with that of the straight tube unit due to the higher natural convection effect by proper utilization of frustum tubes. The study of the HTF pattern shows that where the HTF direction in both the inner and outer tubes are in the gravity direction, the maximum discharging rate can be achieved. For the best configuration, the discharge time is reduced negligibly compared with that for the system with straight tubes which depends on the dimensions of the PCM domain.

## 1. Introduction

Design and development of phase change material (PCM) based thermal energy storage (TES) has been widely attended recently due to the high capacity in latent heat storage without significant temperature changes in the storage unit [1,2]. For the heating and cooling systems in buildings when a uniform temperature is required, PCM-based TES can be more effective [3,4]. However, the main problem of PCM is the weak conductive property of the PCM results in low thermal diffusion inside the PCM [4,5]. Thus, the heat transfer process to or from the PCM is done slowly which reduces the efficiency of the system [6,7,8]. Furthermore, in the heat storage process, this limited characteristic causes high charging time and also a low-performance charging process [9,10]. It means that, during the charging process, the PCM cannot enjoy all the capacity of the heat source. During the discharging process, the heat retrieval rate is not high enough to provide a uniform output temperature during the discharging time which reduces the efficiency of the unit outcome in limited usages of PCM-based heat storage systems [11,12,13].

To solve the drawbacks of PCMs in TES applications, there are different methods which can be categorized into two main groups, i.e., (1) methods that improve the thermophysical properties of the PCM, for instance, the use of nanoparticles inside the PCM [14,15,16,17], (2) methods that increase the thermal rate inside the PCM or from the heat source to the PCM such as adding fins, adding metal foams and modifying the geometry [18,19,20]. There are various works in the literature investigating the thermal improvement inside the PCM-based TES systems using one enhancement method as well as simultaneous usages of various enhancement methods [21,22].

Alizadeh et al. [23] studied a finned triple-tube TES system using V-shaped fins during the discharging process. They add nanoparticles to the PCM to improve the thermal conductivity of the PCM. The outcomes revealed that adding the V-typed fins is more efficient than nanoparticles to improve the discharge rate. Elmaazouzi [24] performed a numerical analysis on the operation of a shell and tube TES unit equipped with annular fins. They showed the importance of fins addition on the performance of the charging process. Moreover, a higher thermal rate was shown using a higher number of fins. Tiari et al. [25] experimentally compared two different types of fins, i.e., annular and radial fins on the performance of a PCM-based TES system in comparison with the no-fined case. In the proposed configurations, radial fins show better performance through both charging and discharging compared with the annular fins. Shen et al. [26] investigated the efficiency of a finned shell and tube PCM-based system considering the effect of thermal radiation. Yang et al. [27] studied a non-uniform annular finned pipe TES and showed that the melting rate can be decreased by almost 63% and the heat storage rate can be improved by almost 85%. Mahdi et al. [28] studied the performance of a horizontal finned double pipe TES equipped with longitudinal fins. They revealed that because of the convection heat transfer, fewer and smaller fins at the upper part with larger fins at the lower part are more effective to shorten the charging rate. Li et al. [29] numerically investigated the performance of multiple PCM units considering different volume ratios and various melting points for the PCM and showed a 32% improvement in the heat storage rate compared with the PCM-only unit. Mosaffa et al. [30] investigated the discharging process in a shell and tube TES unit and showed that the heat transfer rate is higher in the system compared with that using a rectangular shell. Ho and Gao [31] achieved practical work on the influences of nanoparticles in a vertical PCM-based system using alumina as the nanoparticles and n-octadecane as the PCM. Kumaresan et al. [32] examined multi-wall carbon nanotube (MWCNT) on the performance of PCM-based TES systems to improve the heat transfer rate by increasing the thermal conductivity of PCMs. Bazai et al. [33] numerically evaluated to improve the performance of a double pipe PCM-based system using the geometry modification technique. They examined the effects of using an elliptical tube with various aspect ratios, angular positions, and diameters of the inner ellipse. They showed a maximum improvement of 61% in the heat storage rate. Li et al. [34] examined the simultaneous effects of adding metal foam and nanoparticles on the performance of a triple pipe PCM-based TES system using RT35 as the PCM. They also examined the directions of heat transfer fluid inside the heat exchanger. They showed that in the existence of a high conductive porous medium, the impact of nanoparticle addition is insignificant.

Modifying the geometry is one technique to enhance the effectiveness of PCM-based TES heat exchangers without adding any additive to the PCM which can be one advantage of this technique compared with the other enhancement methods [35,36,37]. Mahdi et al. [38] modified a shell and tube heat storage system using multiple PCM combined with other enhancement methods, i.e., metal foam and nanoparticle addition during the solidification process. They showed that the solidification time can be reduced by up to 94% depending on the number of PCM layers and the number of metal foam segments. Shahsavar et al. [39,40,41] studied the performance of double and triple pipe PCM-based heat storage units during melting and solidification using RT35 as the PCM. They modified the geometry using a wavy pipe compared with a straight pipe. They examined the effects of wavelength and wave amplitude using uniform and nonuniform wave distribution and showed the importance of geometry modification on the performance of the phase change process. Wołoszyn et al. [42] studied a novel helical-coiled tube enhanced with spiral fins and a conical shell and showed that the melting time can be reduced by up to 60% compared with a normal TES module. Najim et al. [43] modified the geometry of a vertical triple tube PCM storage system by adding a fin at the bottom of the storage unit during the melting process using RT35 as the PCM. Due to the natural convection effect, the PCM at the bottom melts slower compared with the PCM at the top of the storage unit. In the proposed system, they showed an almost 10% reduction in the melting time and a 9% improvement in the heat storage rate by solving the problem of a lower melting rate at the bottom of the heat storage unit.

During the fixed temperature through the phase change process, PCM based heat storage unit can be very effective in heating systems providing a uniform output temperature during the solidification [44,45,46]. Thus, there are a lot of studies working on the solidification of PCMs using various enhancement techniques. Sardari et al. [47,48,49] numerically developed a composite metal foam/PCM heat exchanger to warm a room using force convection air. The air passed from the middle of the composite PCM to gain heat and solidify the PCM. They showed that by using a suitable flow rate for the heat transfer flow rate, proper design, and employing the heat transfer enhancement technique, uniform output air temperature can be achieved from the TES device. In their study, the size of the heating elements as the heat source was also optimized. Talebizadehsardari et al. [50] studied the consecutive melting and solidification of PCM-based heat storage units. They modified the geometry by employing a zigzag configuration for heat transfer fluid passage to enhance the heat transfer area in the PCM domain. They examined different angles of the zigzag plates and showed almost 33% enhancement in the heat storage rate by increasing the angle of the zigzags. Huang et al. [51] examined the solidification process using different enhancement techniques in a triple pipe PCM-based unit. The effects of a novel configuration of fins addition and the use of multistage inner tubes were examined simultaneously and separately. They showed that the solidification performance can be improved by up to almost 50% for the best position and diameter of inner tubes.

This study aims to improve the solidification performance of a vertical triple pipe PCM-based heat exchanger using different configurations of frustum tubes for the inner, middle, and outer tubes. According to the literature review, by changing the geometry of the heat storage unit, the effect of natural convection can be changed. Thus, this study focuses on the implementation of frustum tubes in a triple tube heat exchanger to enhance the natural convection effect to improve the solidification performance of the system. The effect of heat transfer fluid flow direction is also examined in this study to improve the performance of the system without adding any additive to prevent reducing the volume of PCM. Different flow directions considering gravity direction are examined. The results of this study provide guidelines for a high-performance design of PCM heat exchangers.

## 2. Problem Description

In the current work, a vertical triplex tube equipped with frustum tubes as the inner and middle tubes is examined. Inside, the middle tube is filled with the PCM supported by single or multi-internal frustum tubes to improve the rate of heat transfer during the solidification. The HTF (water) flows across the inner and outer channels. Both straight and frustum tubes are considered for the inner tube where water is passed.

**Figure 1:**
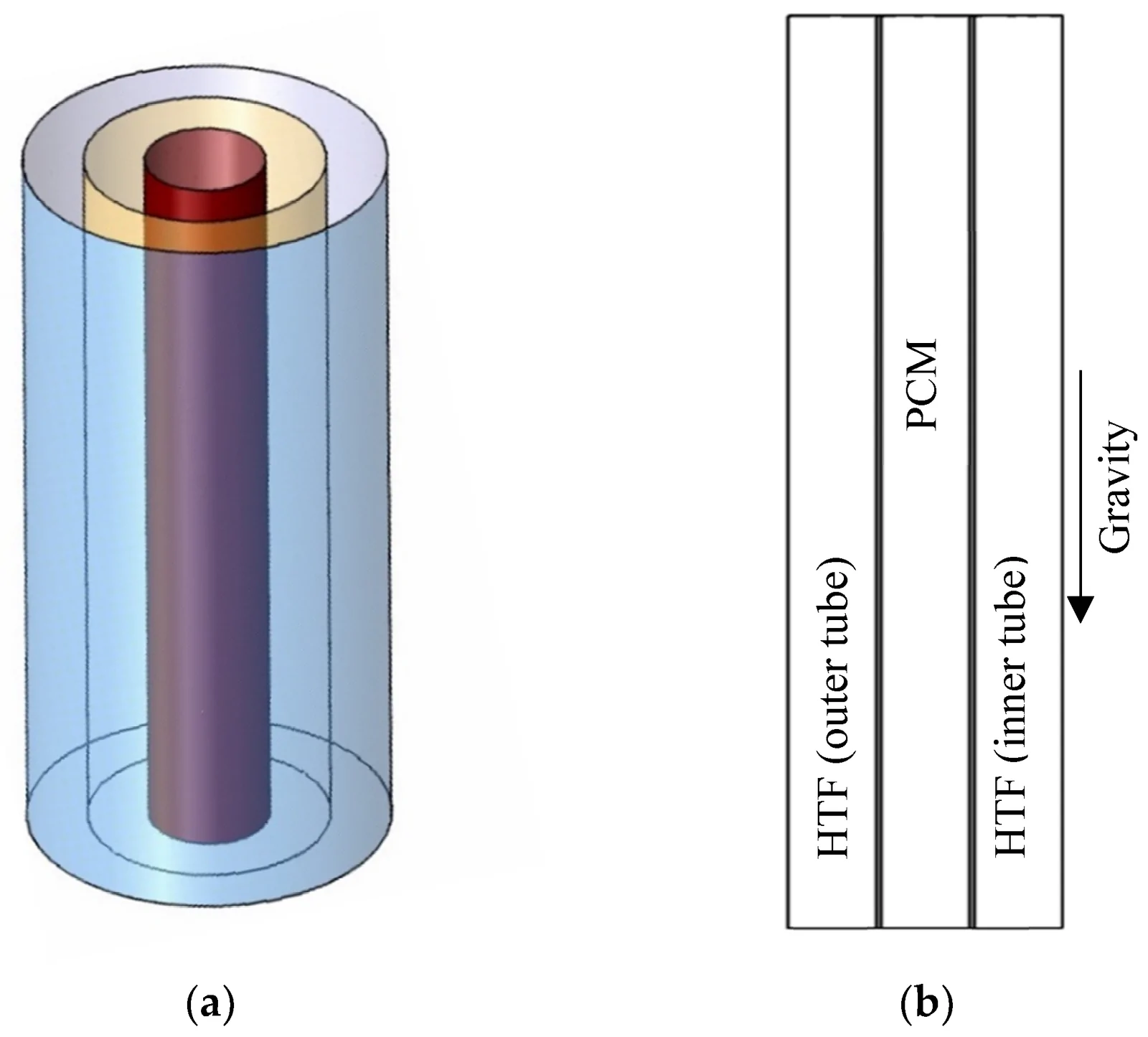
A schematic of the studied triplex tube in (**a**) 3D and (**b**) axisymmetric conditions.

The height of the tube is considered 250 mm. In the system with straight tubes, the hydraulic diameters of the interior, middle, and exterior tubes are considered 20 mm, and the thickness of the interior and exterior channels is also considered 1 mm. The system with a straight tube is shown in Figure 1a. Due to the nature of the heat transfer problem being examined and the scarcity of circumferential flow variation, the system is regarded as axisymmetric, as seen in Figure 1b. The boundary conditions and directions of the HTF flow, as well as gravity, are also illustrated in Figure 1b. The HTF’s input temperature and flow Reynolds number are set to 15 °C and 1000, respectively. The PCM’s initial temperature is also considered 50 °C.

**Figure 2:**
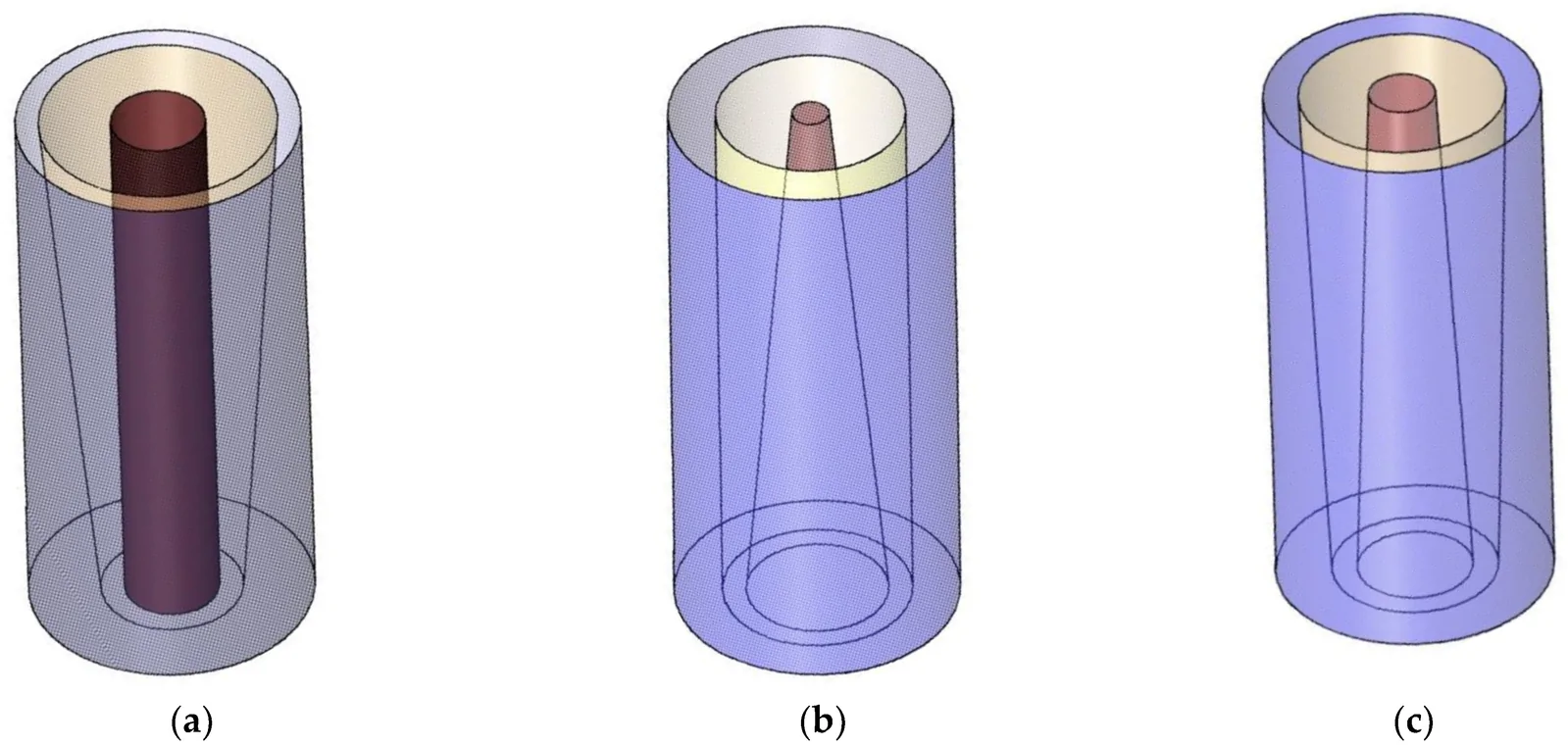
The diagram of the vertical triplex-tube TES system with a frustum tube. (**a**) Changing the middle tube to the frustum; (**b**) changing the inner tube to the frustum; (**c**) changing both the inner and middle tube to the frustum.

In addition to the ordinary straight triplex tubes shown in Figure 1a, there are three more scenarios considered in this study, including changing the middle tube to a frustum tube (Figure 2a), changing the inner tube to a frustum tube (Figure 2b), and changing both the inner and middle tube to frustum tubes (Figure 2c). In addition to the various tube designs, different gap widths are assessed considering the hydraulic diameters of 5, 10, and 15 mm for the middle tube at the bottom of the heat exchanger. The hydraulic diameter of the middle tube at the top of the heat exchanger is then determined considering a constant volume for the PCM equal to the PCM volume in the straight triplex tube heat exchanger. It should be noted that in the case of changing the inner tube to a frustum tube, it is not possible to use 5 mm as the hydraulic diameter of the pipe at the bottom due to the slope of the frustum and dimensions of the system.

In addition, the directions of the HTF flow in the interior and exterior tubes are examined in this study during the solidification. Li et al. [52] studied the direction of HTF flows for the melting mechanism and showed that for the counter-current flow (flow direction is similar to the gravity direction in the inner tube), the highest melting can be achieved; however, it can be varied for the solidification process which is studied in this paper considering four different scenarios as follows:

- co-current (both similar gravity): the flow direction in the inner and outer tubes are in the gravity direction;
- co-current (both opposite gravity): the flow direction in inner and outer tubes are opposite to the gravity direction;
- counter-current (inner similar gravity): the flow direction in the inner tube is the same as gravity;
- counter-current (outer similar gravity): the flow direction in the outer tube is the same as gravity.

**Figure 3:**
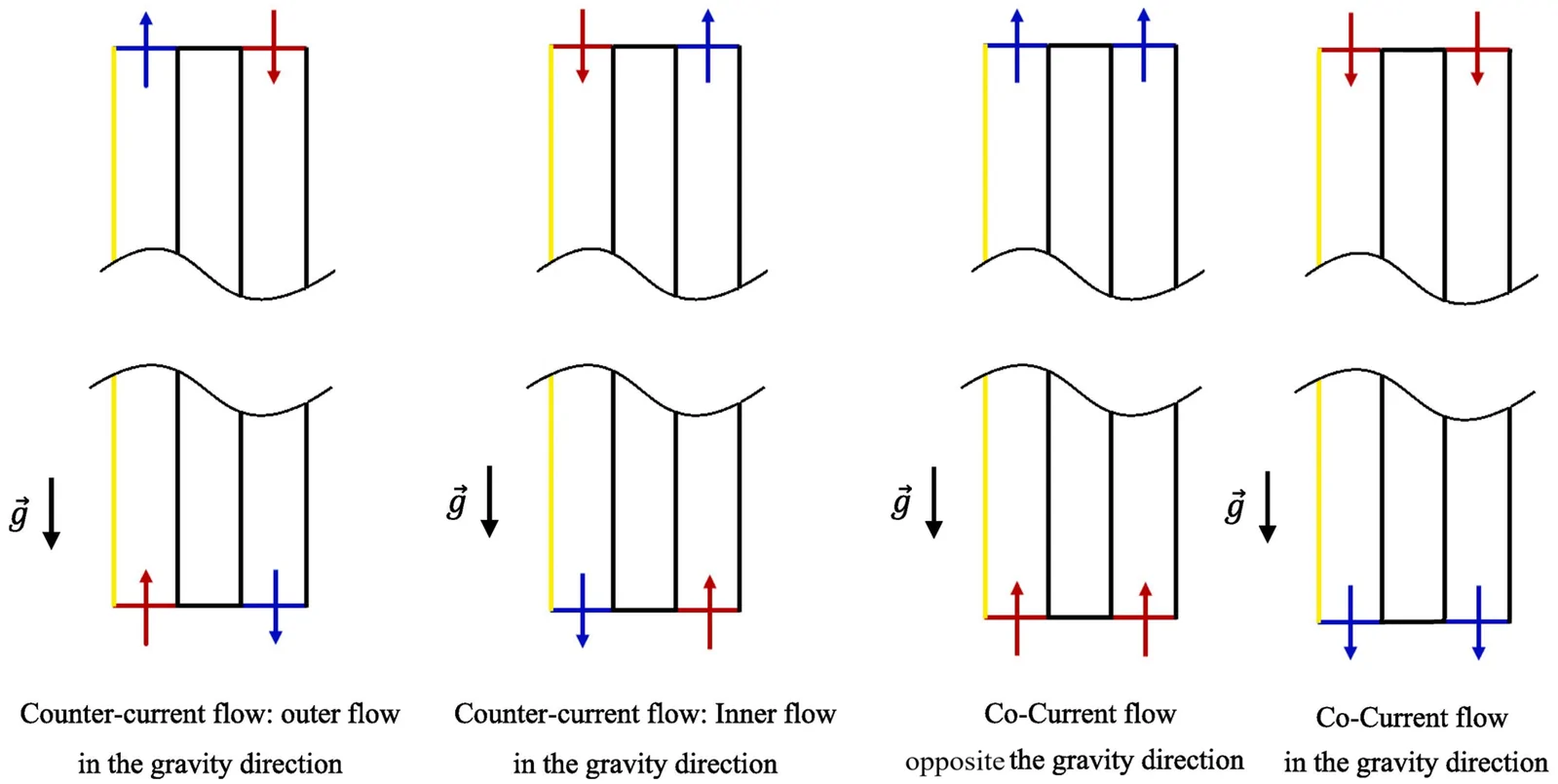
Various fluid flow patterns for the HTF.

The cases are presented in Figure 3.

The utilized PCM is presented in Table 1 which is considered RT-35 which has a suitable melting point for buildings’ applications as well as integrated systems with solar and geothermal energy systems.

## 3. Mathematical Formulation

The simulation of phase changing of the PCM is based on the enthalpy–porosity method developed by Brent et al. [53,54]. In this method, the equal value of the liquid part and the porosity were assumed within each cell of the computational field. To assign the governing formulations, some assumptions are considered [40,41], i.e., (1) utilizing the Boussinesq approximation for buoyant influence changes, (2) considering the stream of molten PCM laminar and incompressible, (3) ignoring thermal loss to the ambient, (4) no-slip boundary conditions at the walls.

The conservation formulations of continuity, momentum, and energy are then expressed as [55]:

(1) $$\frac{\partial \rho }{\partial t}+\nabla \cdot \rho \vec{V}=0$$

(2) $$\rho \frac{\partial \vec{V}}{\partial t}+\rho (\vec{V}\cdot \nabla )\vec{V}=-\nabla P+\mu (\nabla^{2}\vec{V})-\rho \beta \left(T-T_{ref}\right)\vec{g}-\vec{S}$$

(3) $$\frac{\rho C_{p}\partial T}{\partial t}+\nabla (\rho C_{p}\vec{V}T)=\nabla \left(k\nabla T\right)-S_{L}$$

( $\vec{S})$ is indicated as the velocity damping term for the phase change and it disappears at the liquid phase of the PCM and diverges at the solid phase damping the fluid velocity until zero. This term allows to solve the momentum equation in a fixed grid without tracking the location of the interface between the solid and liquid phases which is defined as [56]:

(4) $$\vec{S}=A_{m}\frac{{\left(1-\lambda \right)}^{2}}{\lambda^{3}+0.001}\vec{V}$$

the parameter of the mushy zone ( $A_{m}$ ) equal to 10^5^ based on the literature [57,58]. To evaluate the phase transition progression, λ (the liquid part of PCM) is considered as [59]:

(5) $$\lambda =\frac{\Delta H}{L_{f}}=\left\{\begin{array}{c} 0\;\mathrm{if}\;T<T_{S}\\ 1\;\mathrm{if}\;T>T_{L}\\ \frac{T-T_{S}}{T_{L}-T_{S}}\;\mathrm{if}\;T_{S}<T<T_{L} \end{array}\right\}$$

The Boussinesq approximation is utilized to determine the density variations because of the temperature swipes through the PCM’s phase change progression where density is calculated as [60]:

(6) $$\rho =\rho_{ref}\left(1-\beta \left(T-T_{ref}\right)\right)$$

The source term $S_{L}$ in the energy formula is calculated as follows [61]:

(7) $$S_{L}=\frac{\rho \partial \lambda L_{f}}{\partial t}+\rho \nabla (\vec{V}\lambda L_{f})$$

The thermal energy released rate (solidification rate) through the discharge process is then defined as [62]:

(8) $$\dot{E_{T}}=\frac{E_{end}-E_{ini}}{t_{discharge}}$$

where $t_{discharge}$ is the discharge time and $E_{end}$ and $E_{ini}$ are the energy of the PCM at the end of discharge and initial condition.

## 4. Numerical Description

A combination of the SIMPLE algorithm and Green-Gauss cell-based approach was utilized within the ANSYS-FLUENT solver to assess the heat transfer and fluid flow governing equations of PCM through the phase change procedure. For the momentum and energy formulations, the QUICK differencing technique was utilized with the PRESTO scheme for the pressure correction equations [63]. The convergence basis for terminating the iterative solution is set to be 10^−4^, 10^−4^, and 10^−6^ for the continuity, momentum, and energy formulations, respectively.

The mesh and the time step size independency tests are performed. Therefore, the various meshing of 28,500, 43,000, and 81,620 for the base case (straight tubes) are assessed utilizing the time step size of 0.2 s for the straight triplex unit. Table 2 describes the heat release rate for various cell numbers. As revealed, the outcomes are matching for the meshing of 43,000 and 81,620, and thus, the grid of 43,000 is selected for the next analysis. Table 2 also presents the heat release rate for various sizes of the time step for the nominated mesh. As demonstrated, the outcome data are practically identical for the time step of 0.1, 0.2, and 0.4 s examined, principally for the values 0.2 and 0.1 s. Consequently, 0.2 s is approved as the time step in this study.

**Figure 4:**
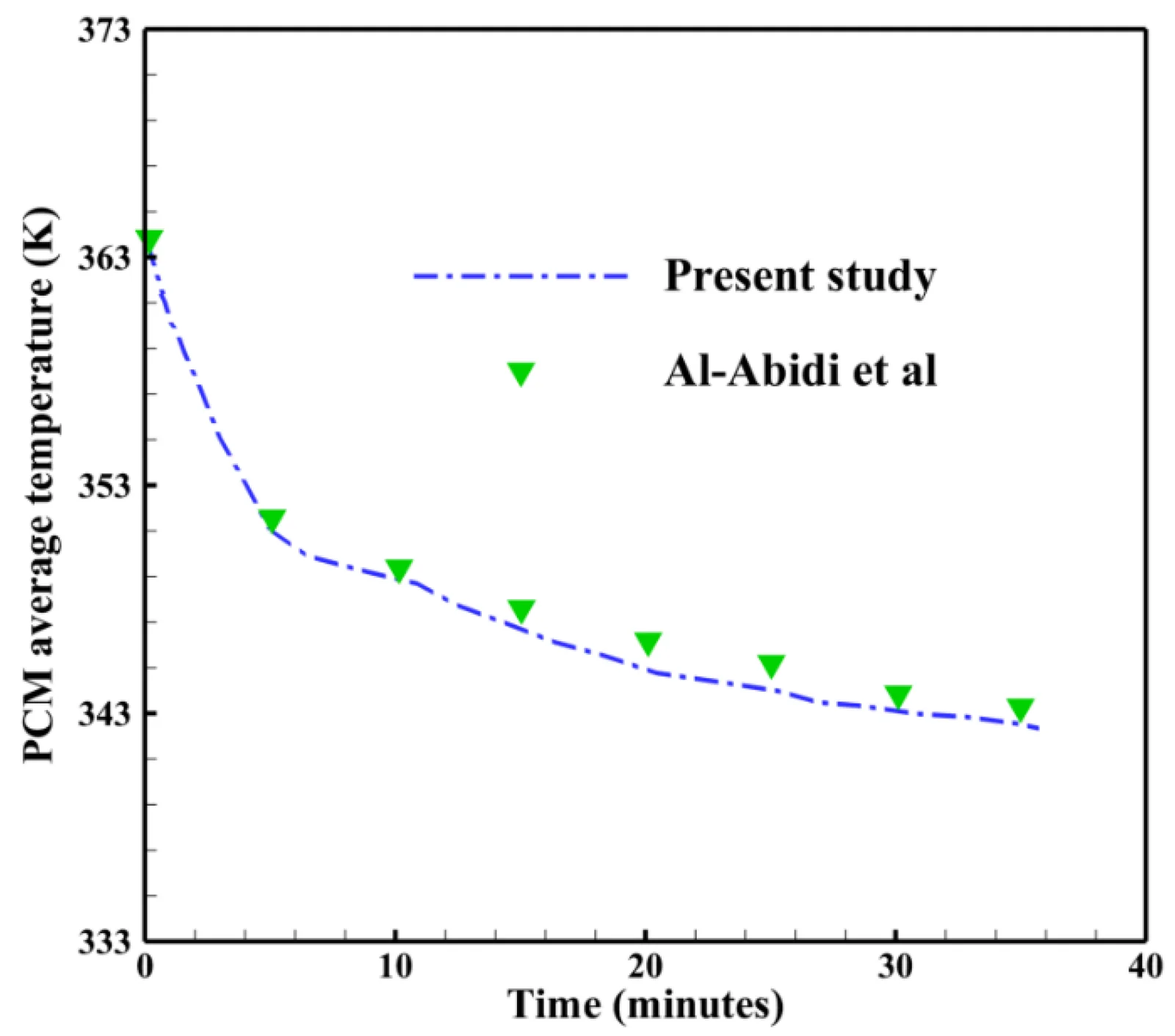
Comparison of the numerical model’s temperature to those of Al-Abidi et al. [59].

To validate the proposed applied model during the solidification process, the results of the current work are compared with the practical study of Al-Abidi et al. [47]. Al-Abidi’s study was a reliable experimental study that, on one hand, has been employed in several studies for validation, and on the other hand, presented all the details of the geometry and PCM used and therefore is suitable for regeneration of the geometry and validation study in this paper [64]. They examined the PCM thermal variation in a triplex-pipe PCM heat exchanger integrated with fins. As noted in Figure 4, there is a good agreement between the PCM mean temperature of the current work and those reported by Al-Abidi et al. [47]. This indicates that the present model accurately predicts the solidification process relative to the experimental data.

## 5. Results and Discussion

### 5.1. The Effect of the HTF Direction

**Figure 5:**
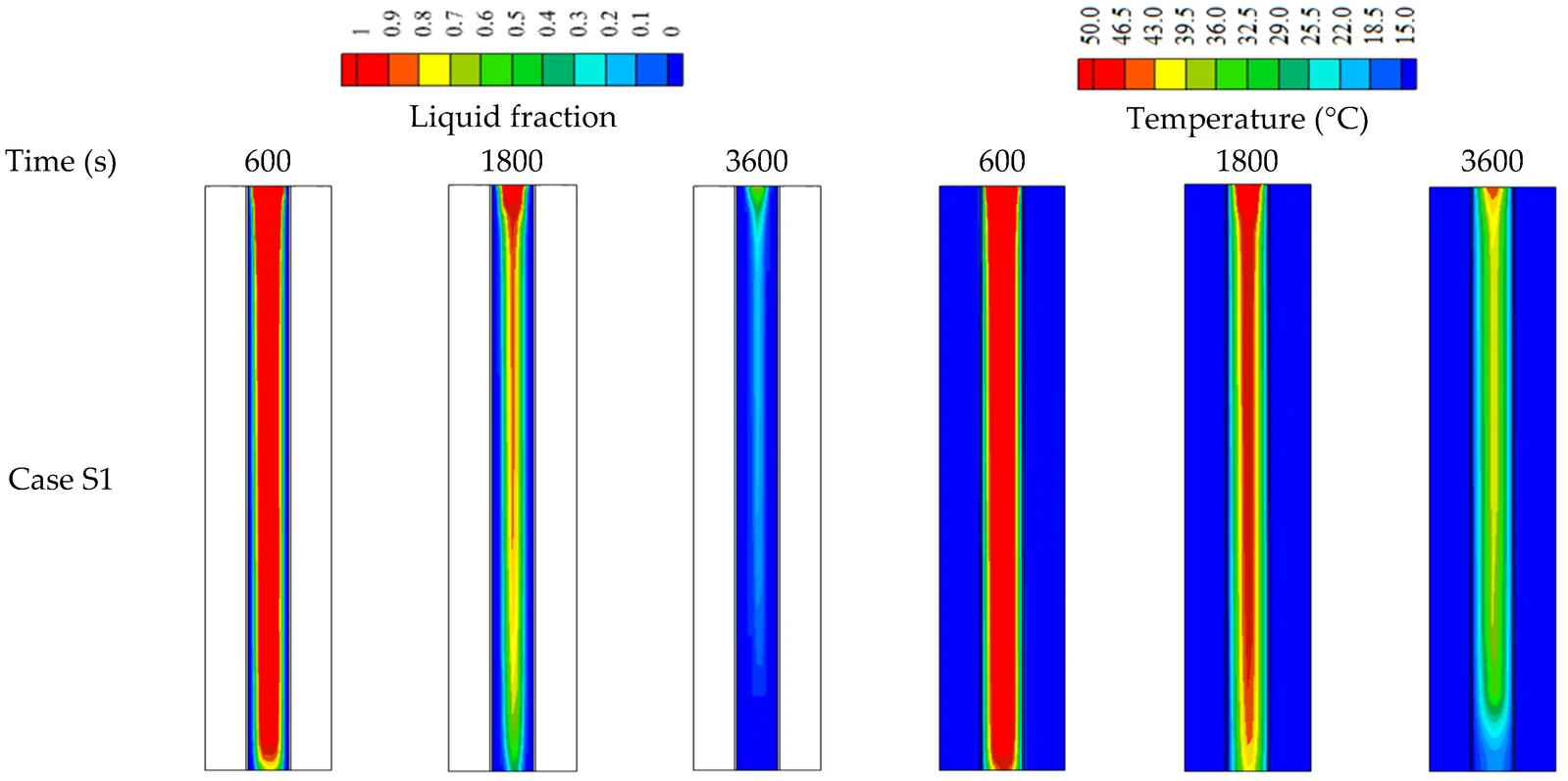

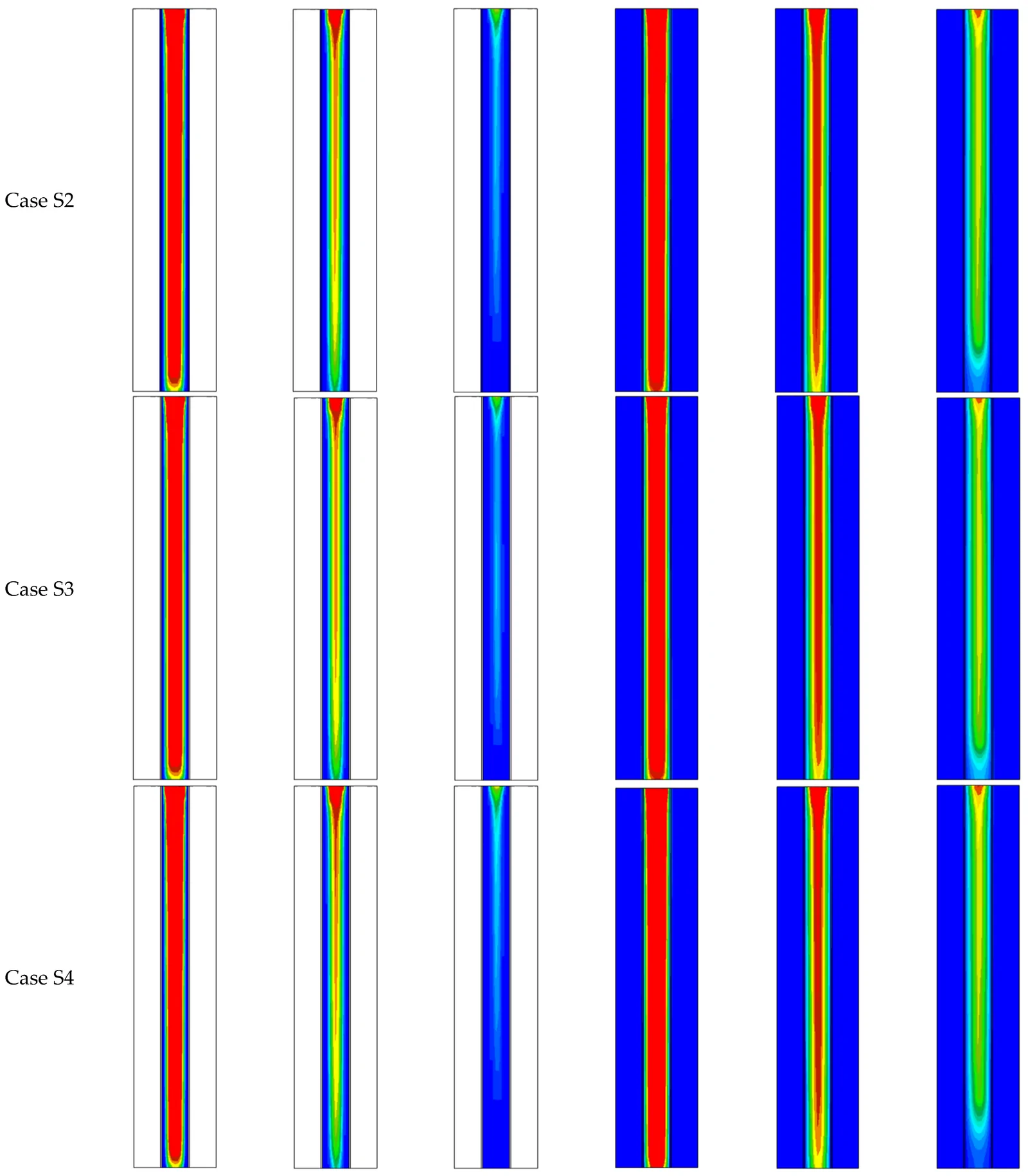
Contours of the temperature distribution for the investigated heat transfer fluid direction over various solidification times.

This section considers the effect of the HTF direction on the phase change process of the PCM. Table 3 shows the conditions of the flow direction of the HTF for cases S1–S4. In these cases, all the tubes are straight. Figure 5 shows the liquid fraction (the three images on the left) and temperature (the three images on the right) contours in different HTF directions. For all the cases, the area adjacent to the HTF channel walls was solidified first due to the convection heat transfer that generates between the wall and the PCM. The solidification process expands gradually as more heat is exchanged in the system. The centreline of the PCM container and the top part are the last regions that solidify due to the far distance from the wall and buoyancy effect, respectively. Within 3600 s, 97% of the PCM solidifies. The solidification process shows almost the same behavior for all the cases, confirming that the direction of the HTF has an inconsiderable effect on the phase change process. The temperature profiles show that the temperature drops in the regions besides the walls, causing a solidification in those areas. The more heat exchange between the PCM and the HTF through the wall, the more dropping in the PCM temperature reveals, consequently, a larger area of solidification. The average temperature of the entire PCM reached 23 °C within 3600 s. The contours of the temperature also indicate that the flow direction has a tiny impact on the phase change process, as the profiles are almost the same for all the cases.

**Figure 6:**
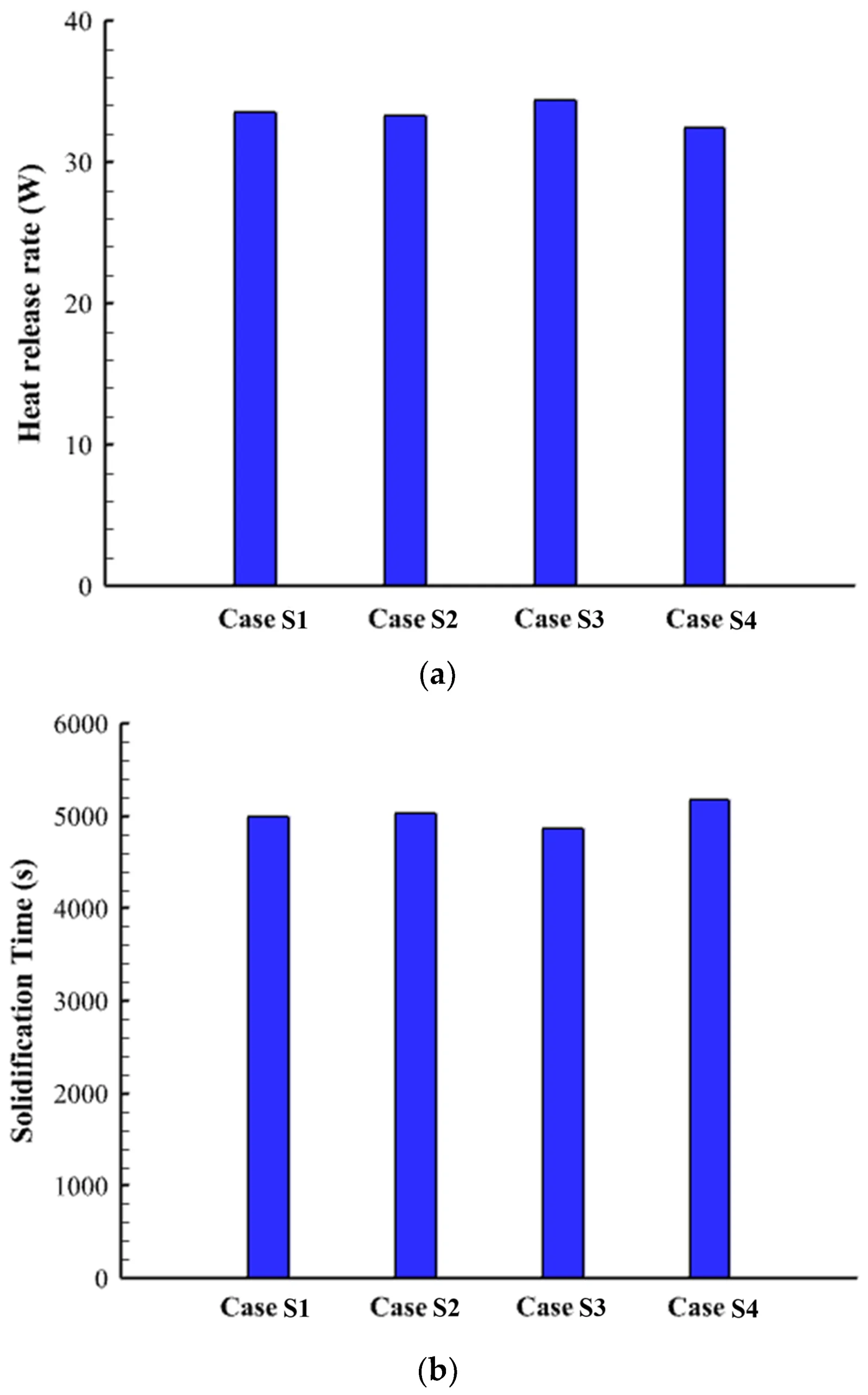
(**a**) Heat release rate, and (**b**) solidification time for the solidification completion for different directions of the heat transfer fluid flow.

Figure 6a,b illustrates the heat transfer rate and the total discharge time for all the cases. The figures show that those parameters are in the same range (with the small differences), whereas, the maximum heat release found in case S3 (HTF is in a gravity direction in both channels), which is 34.39 W, and the minimum value found in case S4 (HTF is in the opposite route of the gravity in both channels), which is 32.49 W. The solidification time in case S3 registered 4873 s, which is lower than the time of the solidification in cases S1, S2, and S4 by 2.7%, 3.4%, and 6.1%, respectively. The only reason that causes these tiny differences is that the flow in the gravity direction gains energy due to the gravity force, and this energy increases the velocity of the HTF downward, consequently enhancing the heat transfer coefficient, then more heat transfers. In case S4, the HTF flows against the gravity, which slightly decreases the velocity of the fluid causing dropping with a tiny value in the heat transfer coefficient.

### 5.2. Effect of the PCM Container Shapes

This section analyzes the PCM container shape effects on the solidification time, temperature distribution, and heat release rate. The width of the PCM enclosure reduces at the upper part and expands at the lower part (with eight different patterns). Worth to be noted is that the sizes of the PCM container are the same for all the cases in this study. Based on the formulated numerical model, different simulations were performed to assess the tube shape modification on the thermal reaction of PCM included in a triplex-tube enclosure geometry during discharging (solidification phase). The tube surface area (exposed to the cooling impact according to the target phase-change method) is improved by applying a single internal frustum tube in a vertical triplex-tube unit, withholding the outer shell unchanged. The frustum tube permits for more adjustment of the tube design over the heat-transfer flow direction, producing a relevant approach for more design conditions. Gap width, which indicates the radial space between the interior and middle channels, was used as the reference length of the PCM area. Various gap widths for the annulus between the inner and the middle tubes were studied to achieve the best shape to analyze the advantage of the shape variation through the phase transition process. Three scenarios were implemented to examine the possible thermal reaction enhancement because of the tube geometry modification. The first scenario is converting the middle tube to a frustum tube, the second scenario is converting the inner tube to the frustum shape, and the third scenario is converting both the inner and the middle to the frustum tubes. Three various gap widths (δ = 5, 10, and 15 mm) were considered for scenarios 1 and 3, and for scenario 2 only two gap widths (δ = 10 and 15 mm) were used (shown in Figure 6). The contour lines for the liquid fraction, temperature, melting time, and heat release rate were used to evaluate the influence of the tube modifications during the solidification process.

**Figure 7:**
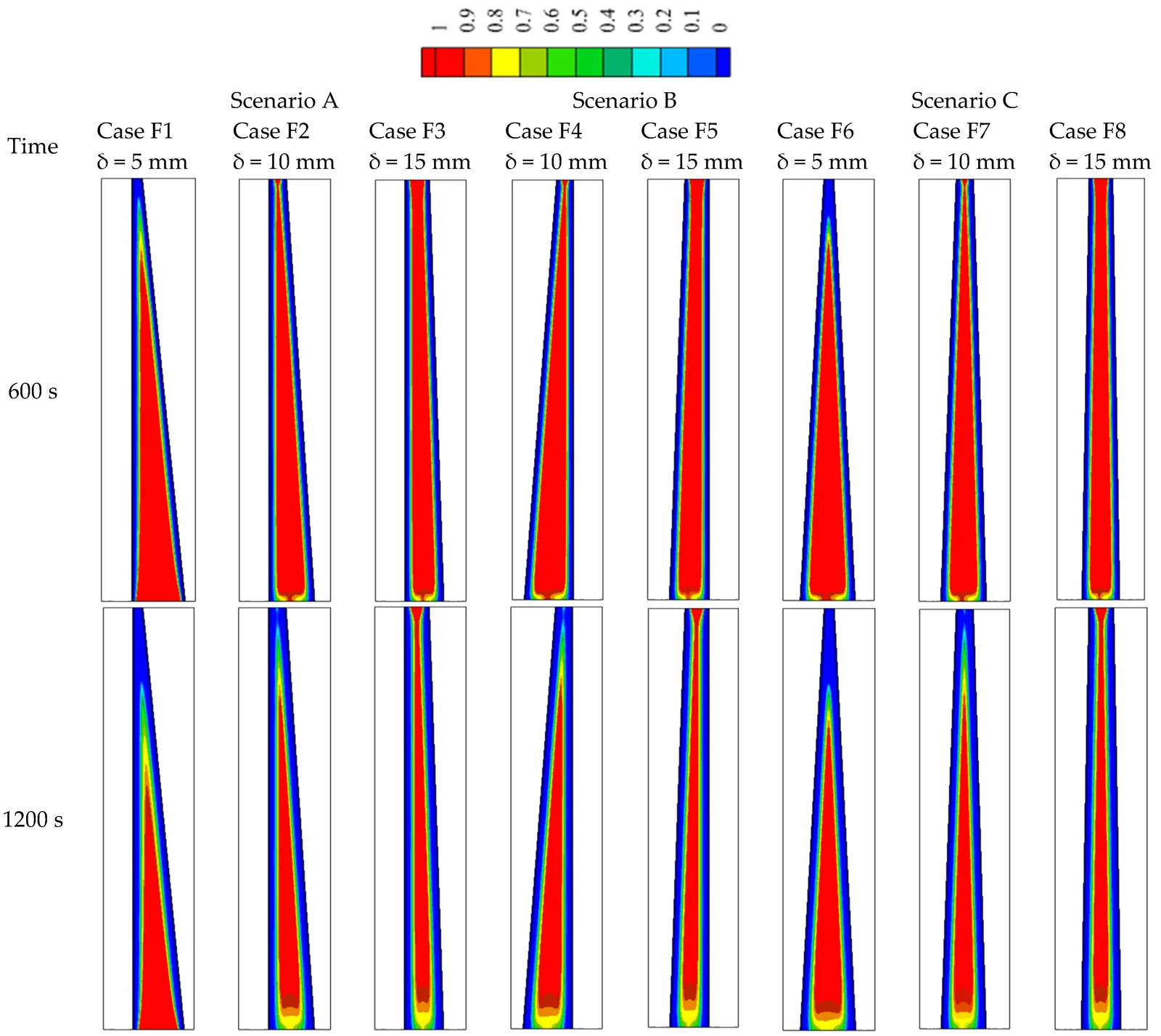

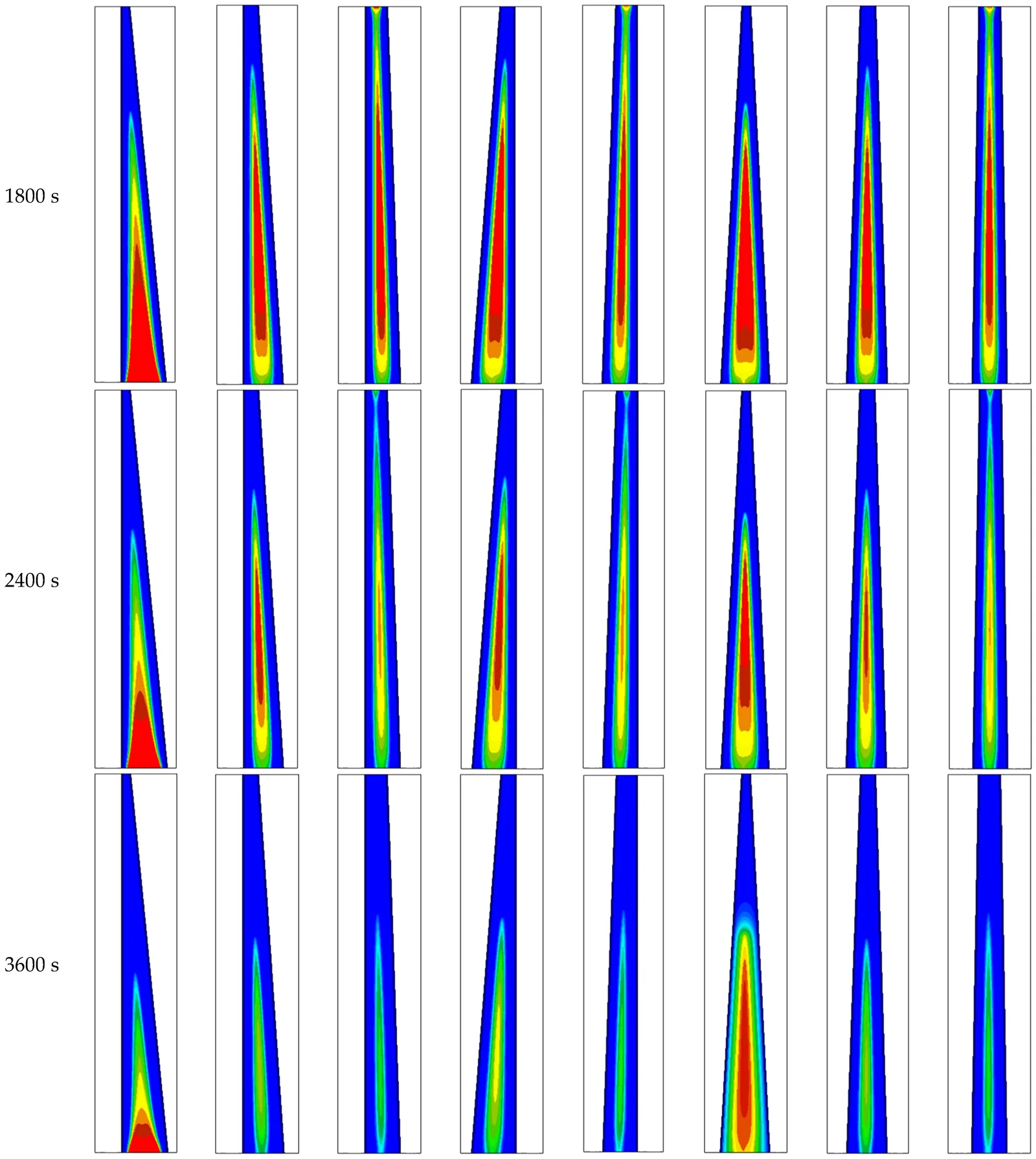

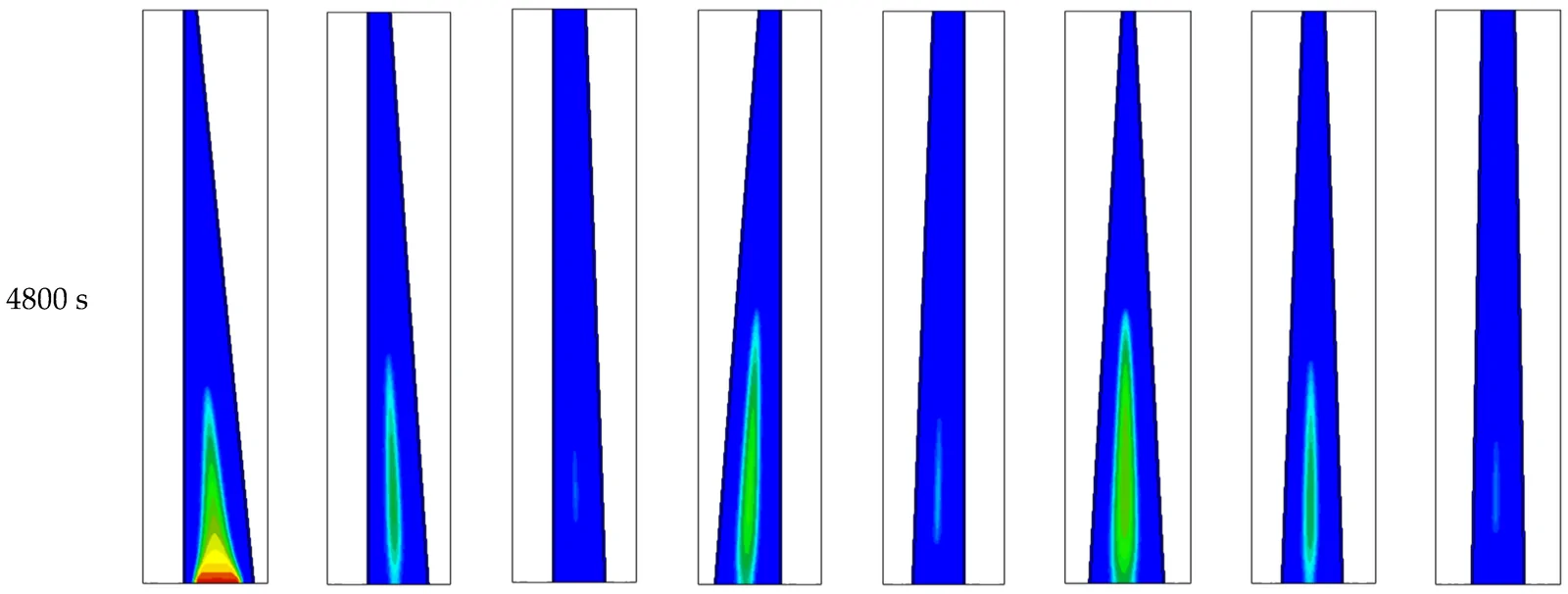
Contours of the liquid fraction for the investigated tube geometries over various solidification times.

Figure 7 shows the liquid-fraction inhalation contours at five different periods of solidification (600, 1200, 2400, 3600, and 4800 s) for the aforementioned scenarios. The convection heat transfer dominates the heat exchanging process initially for (t = 600 s) due to the liquid state of the PCM in the unit. The PCM solidifies at the adjacent areas to the wall, and there are no major differences between the cases. Note that eight cases are studied using frustum tubes called cases F1 to F8. The solidification process expands gradually at (t = 1200 s) due to the heat conduction, which in turn accelerates the discharging process in the upper region of the PCM unit. In addition, the higher temperature differences between the HTF and the PCM in that area play a role to enhance the phase change process. Due to the various densities between the cold and the warm PCM, the solid PCM sink to the bottom part of the domain. In the meantime, the solid part does not collect at the top of the domain, as shown in all the cases in Figure 7. Within 2400 s, the solid part expands more against the liquid PCM, which is shrinking as illustrated through the red zones of the images, especially at the bottom sections. This is caused by the thermal conduction, which launches to dominate the convection heat transfer during the solidification. This leads the solidification process to become faster at the period of 3600 s. During the last period of the discharging process (t = 4800 s), the solid part cover most of the domains except the middle of the bottom section, due to the far distance from the wall (the PCM domain is wider at the bottom than the top section) on the hand and frozen upper part which has a narrow distance between the two walls. However, cases F3, F5, and F8 have almost total solidification due to the relatively narrow distance between the walls at the bottom section. Therefore, the outcome implies that applying a larger gap leads to a smaller lilt angle of the cooling wall, which provides an improved ability to release more heat from the PCM, consequently resulting in faster solidification.

**Figure 8:**
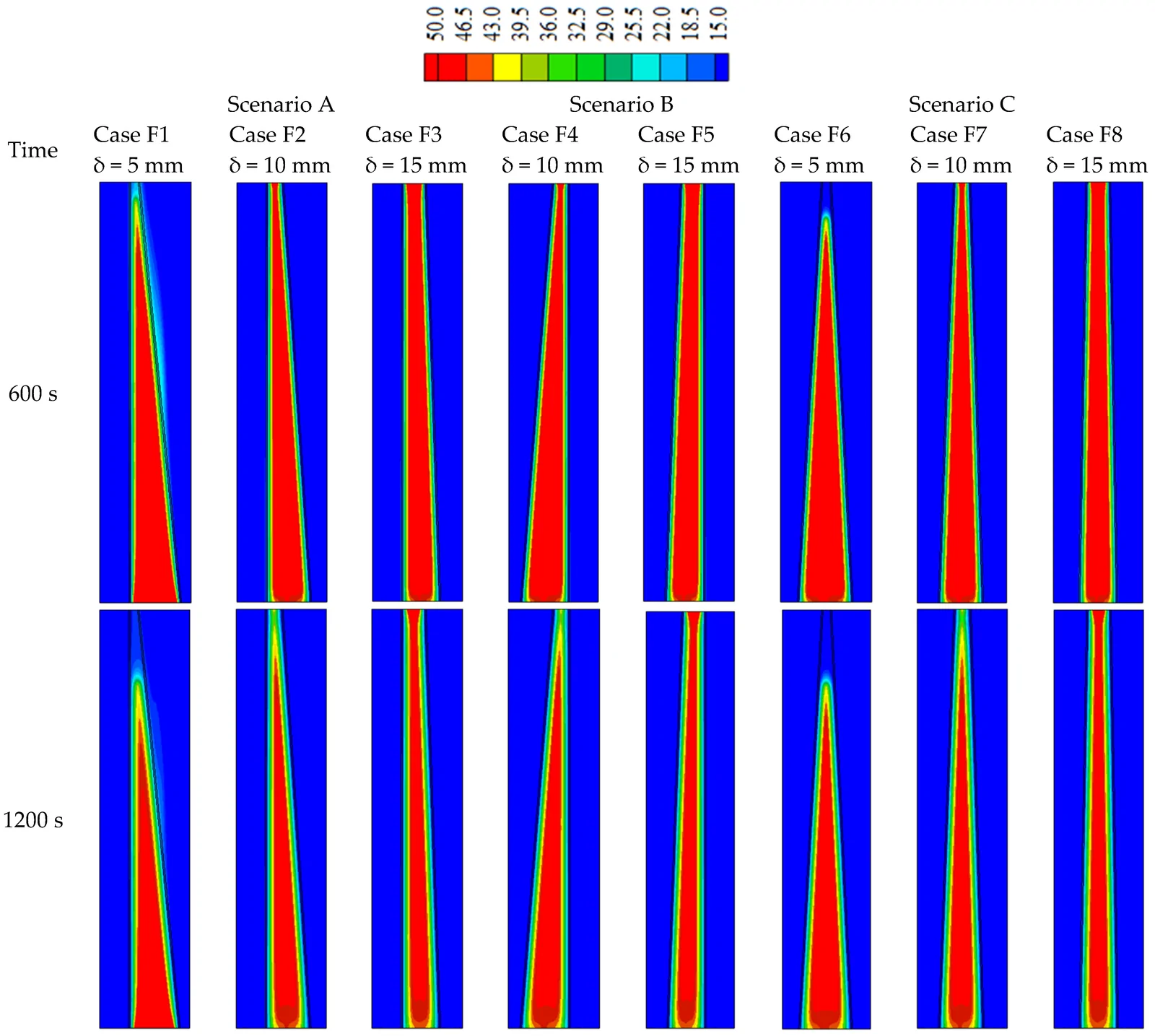

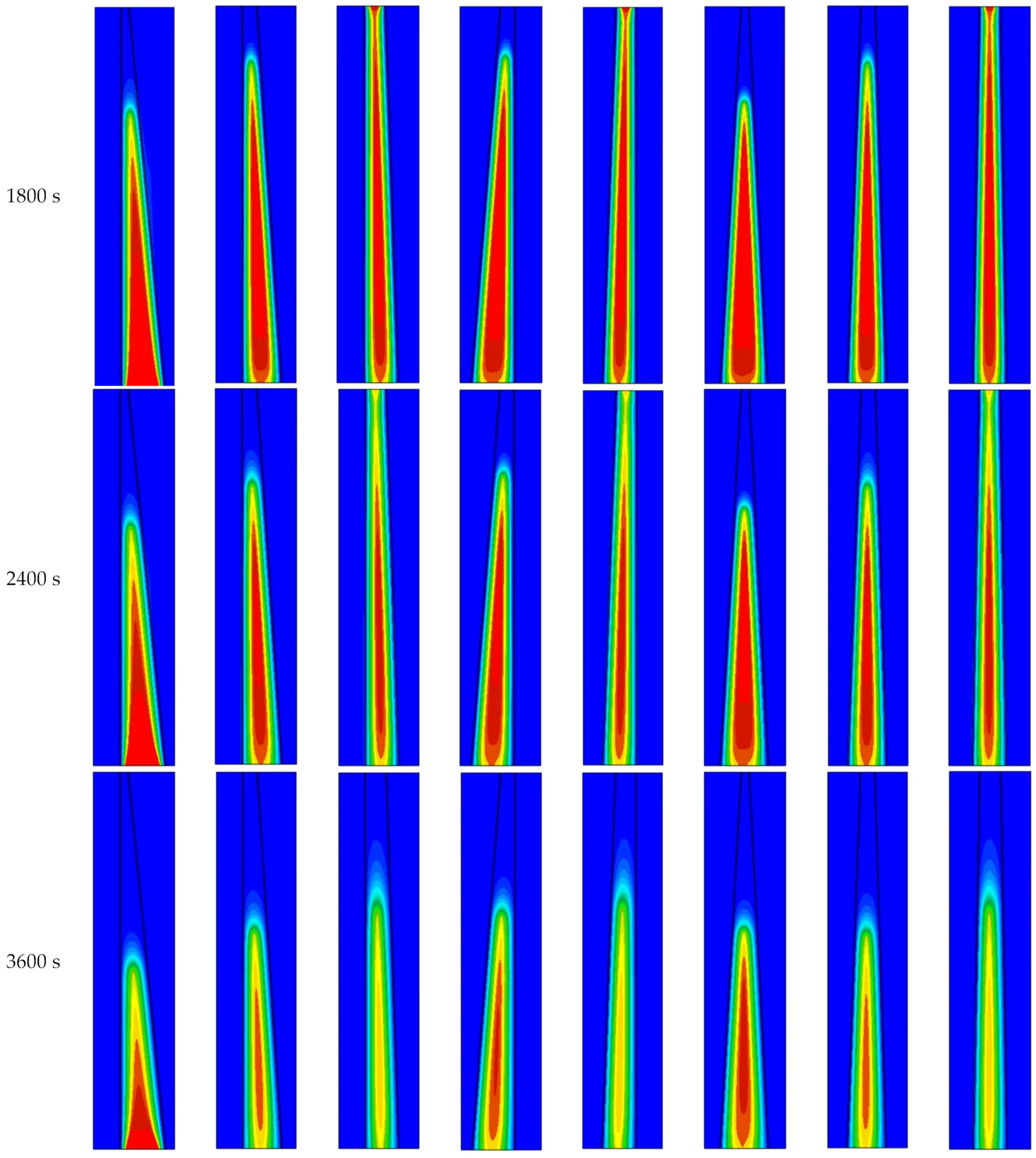

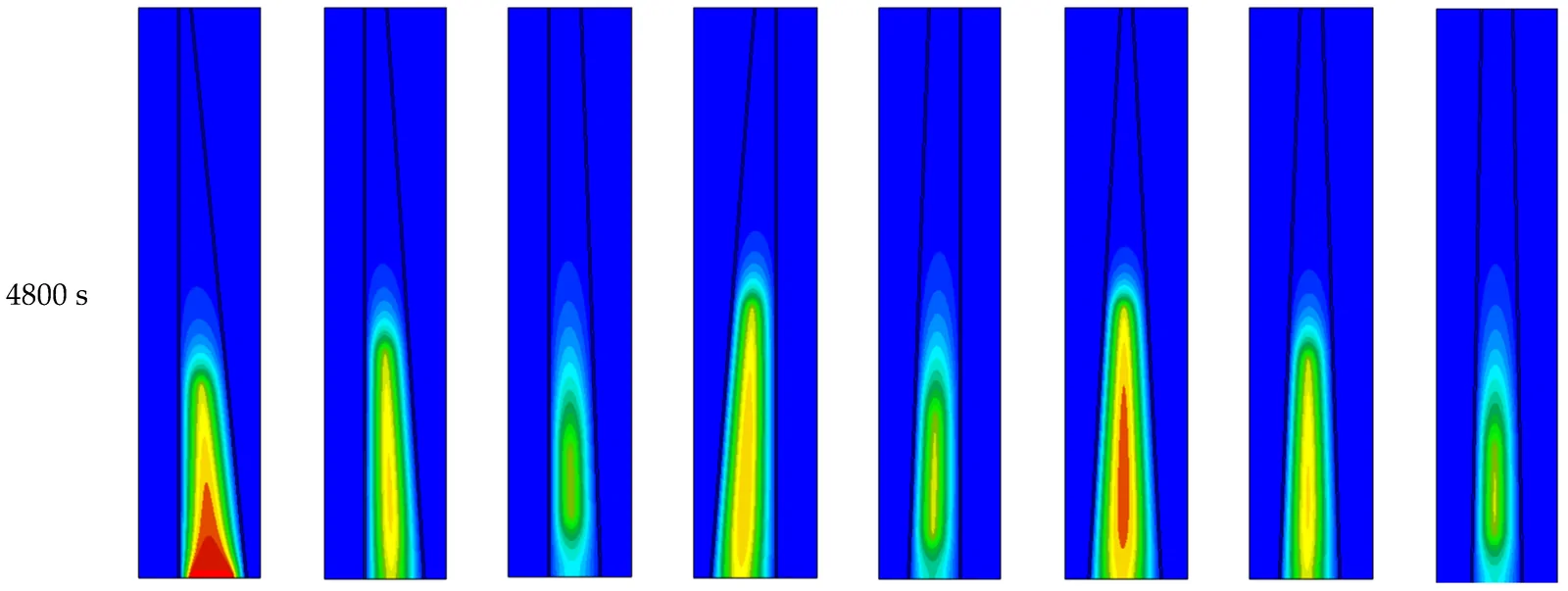
Contours of the temperature distribution for the investigated tube geometries over various solidification times.

Figure 8 illustrates the temperature distribution contours for all the scenarios and cases during various solidification times (600, 1200, 2400, 3600, and 4800 s) in the modified geometries. Up to 1200 s, the inhomogeneity between the conduction and the convection heat transfer over different sections of the PCM causes a drop in the local temperature in the areas beside the walls for all the cases and at the top part in cases F1 and F6. Cases F1 and F6 have a narrow width at the top (δ = 5), which helps the frozen area beside both walls meet and the solidification expands downward to cover a larger area. For all the cases, the solidification starts at the top part, as aforementioned, due to the narrow width of the PCM domain at the top section, and the high-temperature difference between the PCM and the HTF as it flows in the gravity direction (inlet at the top section). The temperature drops more within 2400 s causing more solidification areas. During this process, the buoyancy effect does not show influence due to the frozen area at the top part and confining the solid part between walls at the upper side of the domain. Due to the domination of the conduction effect over the convection heat transfer, and the shorter width at the bottom of the PCM domain in cases F3, F5, and F8 than those in the other cases, the dropping in the temperature steps faster, even in the center of the PCM as shown during 3600 s time. This is confirming that increasing the ratio of the upper to the lower width offers a better performance relatively. Within 4800 s, the average temperature for cases F3, F5, and F8 (δ = 15) are almost the same with a small advantage for case F3, however, cases F1 and F6 (δ = 5) have higher temperatures among all the cases.

**Figure 9:**
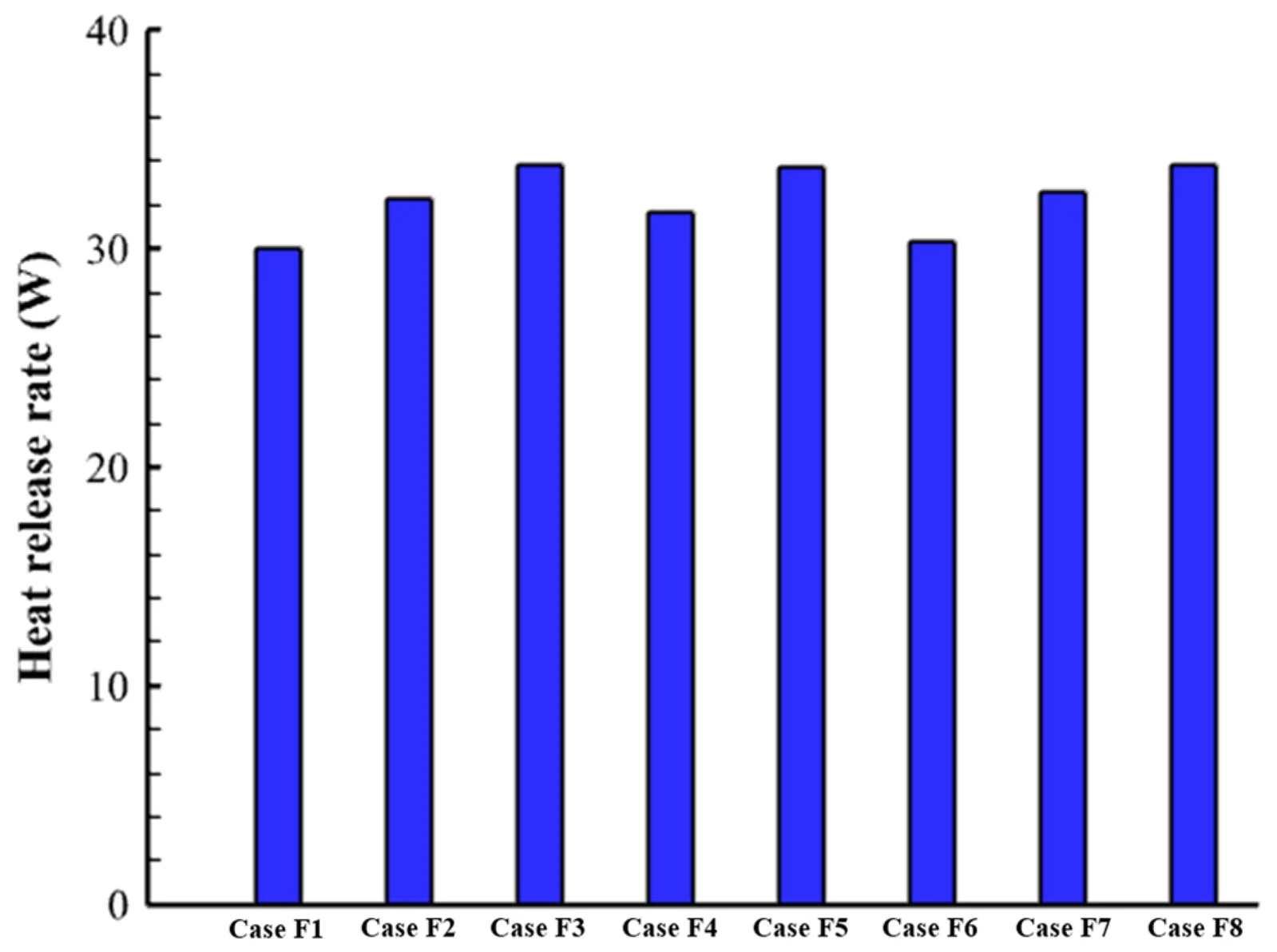
The heat release rate for the solidification completion for different tube configurations (cases F1–F8).

Figure 9 shows the heat release rate for cases F1–F8 at the time of the full solidification state. The figure shows that cases F3, F5, and F8 are the most efficient cases among the others, and case F3 has the highest value. The aforementioned cases have longer width (δ = 15) at the top, making the ratio of the top to the bottom width higher than in the other cases. Reducing this ratio reduces the heat release during the discharge time because of the wide-area at the bottom side, which delays the phase change process at the center of the system. Case F3 has a higher value of the heat release rate with 33.88 W, which is higher than the lowest case (case F1), case F5, and case F8 by 11.5%, 0.3%, and 0.09%, respectively as shown in Table 4. The best configuration among the studied cases in this section regarding the discharging time and heat release is case F3.

**Figure 10:**
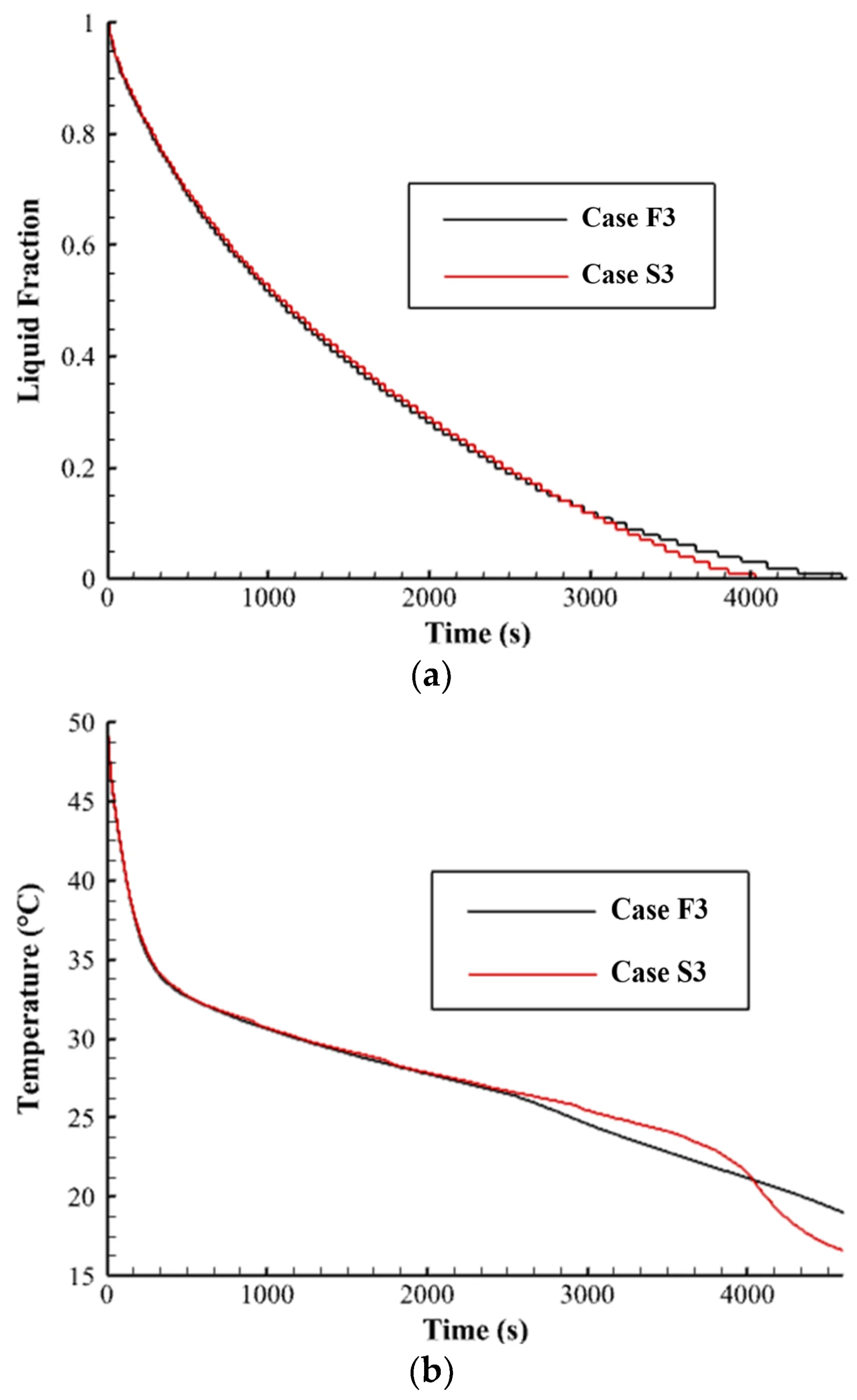
The time-wise difference of (**a**) liquid-fraction and (**b**) mean temperature for the PCM discharging for cases S3 and F3.

The results of cases S3 (straight tube) and case F3 (best case using frustum tubes) based on the total discharge rate and the heat release from the PCM were compared. Figure 10a,b displayed the liquid fraction and temperature variations, respectively, for case F3 compared with case S3. The discharge time and heat storage rate area are presented in Table 5. As shown, using the frustum tube cannot be effective in reducing the discharge time. In case F3, the discharge time is only 10 s less than that for case S3; however, the discharge rate in case S3 is 1.5% less than case S3 during the solidification time. It should be noted that according to Figure 10b, the average PCM temperature for case F3 is lower than that for case S3. Thus, it can be concluded that the results may vary by changing the dimensions of the PCM domain. Moreover, during the solidification process, PCM starts solidifying from the walls and then the discharge process terminates in the center of the PCM domain. Thus, the effect of charging the boundaries from the straight tube to the frustum tube has a negligible effect on the discharge process. By using a frustum tube, the heat transfer area increases between the HTF and PCM which improves the conduction heat transfer at the beginning; however, it suppresses the natural convection effect due to the frustum shape of the PCM domain and thus the effect of frustum tube is low during the discharge process.

## 6. Conclusions

Numerical modeling of the discharge process was carried out to assess the design modification on the thermal management of a vertical triplex tube heat exchanger filled with a PCM. A three-dimensional configuration model was evaluated via commercial software (Ansys Fluent). To improve the efficiency of the cooling processes, internal frustum tubes were integrated into various scenarios compared with the ordinary straight triplex tube system. The effect of flow directions of the HTF was also examined toward the higher performance. Three different scenarios were evaluated including changing the middle tube to the frustum tube, changing the inner tube to the frustum tube, and changing both the inner and middle tube to the frustum tubes. The cases were assessed considering the solidification process duration as well as the heat release rates. The study of flow direction reveals the advantages of the heat exchanger with flowing the HTF in the gravity direction over the other directions of the HTF in the heat exchanger. For the frustum cases, a higher efficiency was found for the cases, which have a longer frustum tube diameter (δ = 15) at the top. Reducing tube diameter reduces the heat release during the discharging time because of the wide-area on the bottom side, which delays the phase change process at the center of the system. Case F3, in which only the middle tube is changed to the frustum tube, has the highest value of the heat release rate among the studied cases equal to 33.92 W, which is higher than the lowest case (case F1), case F5, and case F8 by 11.5%, 0.3%, and 0.09%, respectively. Comparing the best case using the frustum tube with the straight tube system, it was shown that there is a negligible difference between these two cases since the heat transfer mechanism is conduction through the walls which are changed from the straight tube to the frustum tube in this study. However, it can be concluded that changing the dimensions of the PCM domain affects the difference. The temperature in the case of frustum tube usage is lower than that for the system with straight tubes.

## Figures and Tables

**Table 1:** Thermodynamic properties of the PCM used [35].

Properties	$\rho l\;(\mathrm{kg}/m^{3})$	$\rho s\;(\mathrm{kg}/m^{3})$	*L_f_* (kJ/kg)	*C_p_* (kJ/kg·K)	*K* (W/m·K)	*µ* (N·s/m^2^)	*T_L_* (°C)	*T_S_* (°C)	*β* (J/K)
Values	770	860	170	2	0.2	0.023	36	29	0.0006

**Table 2:** Cell number and time step investigation on the heat release rate.

Number of Cells	28,500	43,000			81,620
Time step size (s)	0.2	0.1	0.2	0.4	0.2
Heat release rate (W)	29.41	30.01	29.98	29.89	30.14

**Table 3:** Different directions of the heat transfer fluid flow during the solidification process.

	Inner Tube Inlet	Outer Tube Inlet
Case S1	Gravity direction	Opposite gravity direction
Case S2	Opposite gravity direction	Gravity direction
Case S3	Gravity direction	Gravity direction
Case S4	Opposite gravity direction	Opposite gravity direction

**Table 4:** The heat release rate for the solidification completion for different tube configurations (cases 1–8).

Studied Model	Heat Release Rate (W)
Case F1	29.98
Case F2	32.31
Case F3	33.92
Case F4	31.67
Case F5	33.73
Case F6	30.35
Case F7	32.58
Case F8	33.85

**Table 5:** Heat release rate and discharge time for cases S3 and F3.

Studied Model	Discharge Time	Heat Release Rate (W)
Case S3	4863	33.92
Case F3	4873	34.39

## Data Availability

The data presented in this study are available on request from the corresponding author.

## Nomenclature

$A_{m}$	The mushy zone constant (kg/s m^3^)	**Greek symbols**	
*C*	Inertial coefficient	β	Thermal expansion coefficient (1/K)
$C_{p}$	PCM specific heat (J/kgK)	λ	Liquid fraction
*D*	Hydraulic diameter (m)	α	Thermal diffusivity (m^2^/s)
*E*	Energy (J)	μ	Dynamic viscosity (kg/ms)
g	Gravitational acceleration (m/s^2^)	ρ	Density (kg/m^3^)
K	Thermal conductivity (W/mK)		
L	Latent heat of fusion (J/kg)	**Subscribe**	
m	PCM mass (kg)	ref	Reference
P	Pressure (Pa)	l	Liquid
$\dot{E_{T}}$	Solidification rate (W)	s	Solid
$t_{discharge}$	Discharge time (s)	f	Fraction
T	Temperature (K)	m	Mean
$T_{m}$	Melting point temperature (K)	end	Final
$u_{i}$	Velocity component (m/s)	ini	Initial
$\vec{V}$	Velocity vector (m/s)		
