# Correction: Ju et al. Discharge Enhancement in a Triple-Pipe Heat Exchanger Filled with Phase Change Material. *Nanomaterials* 2022, *12*, 1605

**DOI:** 10.3390/nano16150958

**Published:** 2026-08-04

**Authors:** Yongfeng Ju, Roohollah Babaei-Mahani, Raed Khalid Ibrahem, Shoira Khakberdieva, Yasir Salam Karim, Ahmed N. Abdalla, Abdullah Mohamed, Mustafa Z. Mahmoud, Hafiz Muhammad Ali

**Affiliations:** 1 Faculty of Electronics Information Engineering, Huaiyin Institute of Technology, Huai’an 223003, China; 2 Department of Chemical Engineering, Brunel University London, Kingston Lane, Uxbridge UB8 3PH, UK; 3 Department of Medical Instrumentation Engineering Techniques, Al-Farahidi University, Baghdad 10015, Iraq; 4 Department of Scientific Affairs, Tashkent State Pedagogical University Named after Nizami, Tashkent 100185, Uzbekistan; 5 Department of Pharmacy, Al-Manara College for Medical Sciences, Maysan 62001, Iraq; 6 Department of Pharmacy, Al-Nisour University College, Baghdad 10081, Iraq; 7 Research Centre, Future University in Egypt, New Cairo 11745, Egypt; 8 Department of Radiology and Medical Imaging, College of Applied Medical Sciences, Prince Sattam Bin Abdulaziz University, Al-Kharj 11942, Saudi Arabia; 9 Faculty of Health, University of Canberra, Canberra 2600, Australia; 10 Mechanical Engineering Department, King Fahd University of Petroleum and Minerals, Dhahran 31261, Saudi Arabia; 11 Interdisciplinary Research Center for Renewable Energy and Power Systems (IRC-REPS), King Fahd University of Petroleum and Minerals, Dhahran 31261, Saudi Arabia

In the original publication [1], there were mistakes in the References 7, 8, 10, 13, 17, 21, 22, 37, 45 and 46. The references have now been corrected as follows:

7. Zhang, Q.; Luo, Z.; Guo, Q.; Wu, G. Preparation and thermal properties of short carbon fibers/erythritol phase change materials. *Energy Convers. Manag.* **2017**, *136*, 220–228.

8. Zou, D.; Ma, X.; Liu, X.; Zheng, P.; Hu, Y. Thermal performance enhancement of composite phase change materials (PCM) using graphene and carbon nanotubes as additives for the potential application in lithium-ion power battery. *Int. J. Heat Mass Transf.* **2018**, *120*, 33–41.

10. Wang, K.; Zhang, T.; Wang, T.; Xu, C.; Ye, F.; Liao, Z. Microencapsulation of high temperature metallic phase change materials with SiCN shell. *Chem. Eng. J.* **2022**, *436*, 135054. https://doi.org/10.1016/j.cej.2022.135054.

13. Sharma, A.; Tyagi, V.V.; Chen, C.R.; Buddhi, D. Review on thermal energy storage with phase change materials and applications. *Renew. Sustain. Energy Rev.* **2009**, *13*, 318–345.

17. Hayat, M.A.; Ali, H.M.; Janjua, M.M.; Pao, W.; Li, C.; Alizadeh, M. Phase change material/heat pipe and Copper foam-based heat sinks for thermal management of electronic systems. *J. Energy Storage* **2020**, *32*, 101971.

21. Hassan, F.; Jamil, F.; Hussain, A.; Ali, H.M.; Janjua, M.M.; Khushnood, S.; Farhan, M.; Altaf, K.; Said, Z.; Li, C. Recent advancements in latent heat phase change materials and their applications for thermal energy storage and buildings: A state of the art review. *Sustain. Energy Technol. Assess.* **2022**, *49*, 101646.

22. Zhang, C.-W.; Fan, Y.-B.; Yu, M. Performance evaluation and analysis of a vertical heat pipe latent thermal energy storage system with fins-copper foam combination. *Appl. Therm. Eng.* **2020**, *165*, 114541.

37. Zhang, P.; Xiao, X.; Ma, Z.W. A review of the composite phase change materials: Fabrication, characterization, mathematical modeling and application to performance enhancement. *Appl. Energy* **2016**, *165*, 472–510.

45. Zhang, H.L.; Baeyens, J.; Degrève, J.; Pitié, F. Latent Heat Storage with Phase Change Materials (PCMs). *J. Technol. Innov. Renew. Energy* **2013**, *2*, 340–351.

46. Du, J.; Nie, B.; Zhang, Y.; Du, Z.; Wang, L.; Ding, Y. Cooling performance of a thermal energy storage-based portable box for cold chain applications. *J. Energy Storage* **2020**, *28*, 101238.

The authors state that the scientific conclusions are unaffected. This correction was approved by the Academic Editor. The original publication has also been updated.
